# Generative artificial intelligence and the personalization of health professional education: A narrative review

**DOI:** 10.1097/MD.0000000000038955

**Published:** 2024-08-02

**Authors:** Mohammed Almansour, Fahad Mohammad Alfhaid

**Affiliations:** a Department of Medical Education, College of Medicine, King Saud University, Riyadh, Saudi Arabia; b Department of family and community medicine, College of medicine, Majmaah University, Majmaah, Saudi Arabia.

**Keywords:** continuous professional development, diversity in AI development, educational framework, ethical AI integration, generative artificial intelligence, health professional education, precision medicine

## Abstract

This narrative review examined the intersection of generative artificial intelligence (GAI) and the personalization of health professional education (PHE). This review aims to the elucidate the current condition of GAI technologies and their particular uses in the field of PHE. Data were extracted and analyzed from studies focusing on the demographics and professional development preferences of healthcare workers, the competencies required for personalized precision medicine, and the current and potential applications of artificial intelligence (AI) in PHE. The review also addressed the ethical implications of AI implementation in this context. Findings indicated a gender-balanced healthcare workforce with a predisposition toward continuous professional development and digital tool utilization. A need for a comprehensive educational framework was identified to include a spectrum of skills crucial for precision medicine, emphasizing the importance of patient involvement and bioethics. AI was found to enhance educational experiences and research in PHE, with an increasing trend in AI applications, particularly in surgical education since 2018. Ethical challenges associated with AI integration in PHE were highlighted, with an emphasis on the need for ethical design and diverse development teams. Core concepts in AI research were established, with a spotlight on emerging areas such as data science and learning analytics. The application of AI in PHE was recognized for its current benefits and potential for future advancements, with a call for ethical vigilance. GAI holds significant promise for personalizing PHE, with an identified need for ethical frameworks and diverse developer teams to address bias and equity in educational AI applications.

## 1. Introduction

Within the past half a decade, artificial intelligence (AI) has been driving force across different facets of human lives.^[1]^ The advent of AI has primarily been possible due to the large advancements in technology. This has helped the creation of supercomputers which are then further made possible by the technological leaps taken in terms of quantum computing. This was made possible by a large number of silicon arrays can be placed on a system and this miniaturization has led to the observed increase in computing power resulting in the AI boom.^[2]^

**Figure 1.**
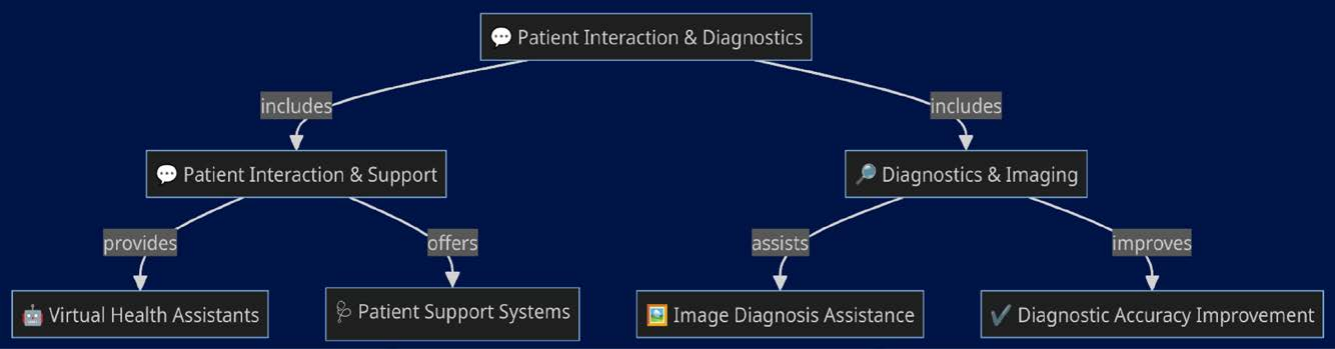
Patient diagnostics and interaction improvements using AI. AI = artificial intelligence.

AI-driven patient interaction tools, such as virtual health assistants, offer support and can manage routine inquiries, which may increase efficiency^[2]^ (Fig. 1). However, these systems can sometimes lack the empathy and deep understanding that human practitioners provide, and they can struggle with more nuanced patient needs. In diagnostics, AI assists with imaging analysis, contributing to more accurate diagnoses in some cases.^[3]^ The technology, though, is supplementary and works best under the guidance of trained radiologists, as it can sometimes produce false positives or miss subtleties in images.^[2,4–6]^

**Figure 2.**
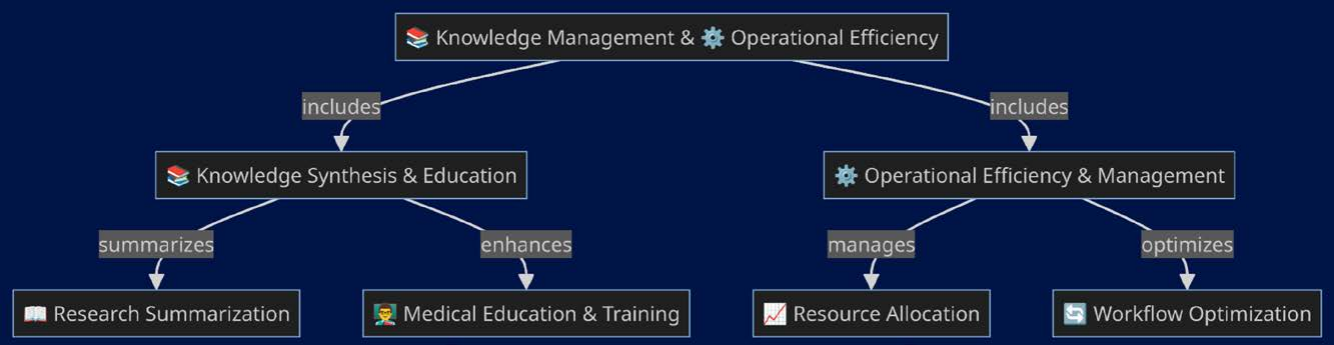
Impact of AI on knowledge management and operational efficiency. AI = artificial intelligence.

AI’s application in synthesizing medical knowledge and aiding education helps healthcare professionals keep up with the latest research. It offers summaries of complex studies, though these summaries may oversimplify or omit critical information, necessitating thorough review by experts.^[7]^ Personalized medicine, supported by AI, attempts to tailor treatment plans to the individual based on algorithms. While this personalization is a step towards more targeted care, it is dependent on the quality and quantity of data available, which can limit its effectiveness^[8]^ (Fig. 2). Operational efficiency in healthcare settings benefits from AI in resource allocation and workflow optimization. The technology can help optimize schedules and manage supplies, but it cannot completely replace human decision-making, especially in unpredictable or novel situations.^[9]^

Large language models (LLMs) and generative artificial intelligence (GAI) are the more recent progressions that have been the result of this leap.^[1,2]^ LLMs and GAI have been incorporated into various facets of the healthcare industry, each serving a distinct function with varying degrees of impact.^[10]^ In the domain of healthcare documentation and electronic health records (EHRs), these technologies facilitate the automatic generation of clinical notes. While this automation reduces manual input and can potentially minimize errors, it is not without challenges, including the need for rigorous oversight to ensure accuracy and relevance.^[11]^ Integration with EHR systems has streamlined data management to a degree, but interoperability issues and concerns about patient privacy persist. The drug discovery and development process has seen AI applied in molecule screening, aiding in the identification of viable drug candidates.^[12]^ This process, while faster, does not guarantee success and must be complemented by traditional research and development methods. AI’s role in speeding up preclinical research offers promise, but the complexity of biological systems often requires a level of nuance that AI has yet to fully replicate.^[13]^

In a broader context, AI describes systems or robots that can accomplish tasks in a way that mimics human intelligence and may iteratively improve themselves based on the data they gather.^[7]^ AI is now widely used in healthcare, particularly in patient interaction, where devices like virtual health assistants handle common questions to boost productivity. AI helps imaging analysis in diagnostics, improving diagnosis accuracy under the guidance of qualified radiologists.^[9]^ AI also makes it easier to synthesize medical information, which helps medical practitioners stay up to date on the most recent findings and operationalize personalized medicine by using data-driven treatment plans.^[2]^

LLMs, generative adversarial networks (GANs), and variational autoencoders (VAEs) are some of the technologies used in GAI, a highly specialized area of AI.^[2,3]^ Based on available data sets, these models are excellent at producing new material, ranging from text to multimedia. The capabilities of GAI are very helpful in the field of medical education for creating adaptive learning systems that modify course material in response to changing requirements for health professionals.^[8]^ Based on student performance and learning pace, these systems can dynamically modify the course materials and exams, enabling a more individualized learning experience.^[11–14]^

This review outlines the present condition of generative AI technologies and their particular uses in the field of personalized health education (PHE). We have also tried to present the basic theories behind customized learning and how GAI can put these concepts into practice to create educational activities that adjust immediately to students’ advancements. Additionally, the review will look into proof of how well generative AI tools work for education and point out where in the literature we need more studies.

## 2. Materials and methods

### 2.1. Review design

The review process commenced with the identification phase, wherein a comprehensive search was conducted across several databases using presearch strings tailored to each database’s indexing system, utilizing a combination of Boolean operators and Medical Subject Headings (MeSH) keywords. This search culminated on the cutoff date of November 2023 (see Table 1 for the details). *Selection criterion:* see the details in Table 2.

**Table 1:** Search strings utilized across the databases.

Database	Search string
PubMed	(“Generative Artificial Intelligence”[MeSH Terms] OR “Generative AI”[Title/Abstract]) AND (“Education, Medical”[MeSH Terms] OR “Health Professional Education”[Title/Abstract]) AND (“Personalization”[MeSH Terms] OR “Customization”[Title/Abstract]) AND “2023/11”[Date - Publication]
Embase	(“generative artificial intelligence”/exp OR “generative AI” OR “generative deep learning”) AND (“education, medical”/exp OR “health professional education” OR “medical training”) AND (“personalization”/exp OR “customization”) AND “202311”:[pub-date]
Cochrane Library	TITLE-ABS-KEY (“Generative Artificial Intelligence” OR “Generative AI” OR “Generative Deep Learning”) AND (“Education, Medical” OR “Health Professional Education”) AND (“Personalization” OR “Customization”) AND (Publication Date: Before 2023-11)
PsycINFO	(“Generative Artificial Intelligence”[MeSH] OR “Generative AI”[Title/Abstract] OR “Generative Deep Learning”[Title/Abstract]) AND (“Education, Medical”[MeSH] OR “Health Professional Education”[Title/Abstract]) AND (“Personalization”[MeSH] OR “Customization”[Title/Abstract]) AND PY=(*-2023)
Web of Science	TS = ((“Generative Artificial Intelligence” OR “Generative AI” OR “Generative Deep Learning”) AND (“Education, Medical” OR “Health Professional Education” OR “Medical Training”) AND (“Personalization” OR “Customization”)) TimeSpan = All Years. Index = SCI-EXPANDED, SSCI. Timespan = Up to November 2023
Scopus	TITLE-ABS-KEY ((“Generative Artificial Intelligence” OR “Generative AI” OR “Generative Deep Learning”) AND (“Education, Medical” OR “Health Professional Education” OR “Medical Training”) AND (“Personalization” OR “Customization”)) AND PUBYEAR < 2024
IEEE Xplore	((“Generative Artificial Intelligence” OR “Generative AI” OR “Generative Deep Learning”) AND (“Medical Education” OR “Health Professional Education” OR “Medical Training”) AND (“Personalization” OR “Customization”)) AND (“Publication Year”: Before 2023)

**Table 2:** Selection criteria devised for this review.

Criterion	Inclusion/Exclusion	Description	Justification
Study design	Inclusion	Peer-reviewed empirical studies, including qualitative, quantitative, and mixed-methods research	To ensure the review was based on robust evidence, studies included were required to have a clear empirical foundation
	Exclusion	Editorials, commentaries, opinions, and reviews	These articles often contained expert opinion or summary but lack primary data, which was necessary for an empirical evidence review
Population	Inclusion	Studies involving health professional education	The focus of the review was on health professional education; therefore, the population of interest is clearly defined
	Exclusion	Studies not specific to health professionals or their education	To maintain the specificity of the review’s context and ensure relevance to the target audience
Intervention	Inclusion	Studies involving generative artificial intelligence applications	The review specifically aimed to assess the role of generative AI in personalizing health professional education
	Exclusion	Studies focusing on non-generative AI technologies or without AI components	Non-generative AI studies were outside the scope of this review, which centers on generative AI capacities
Outcome	Inclusion	Studies examining the personalization of education and training outcomes	The impact of generative AI on personalization was the core interest of the review, making related outcomes crucial
	Exclusion	Studies with unrelated outcomes to education or personalization	Outcomes not related to the personalization of education did not meet the review’s objective and were thus excluded
Publication date	Inclusion	Studies published up to November 2023	This ensured the review is current and incorporates the most recent studies up to the predetermined cutoff date
	Exclusion	Publications before the year 2013	Older publications may not reflect the current state of generative AI technology in education
Language	Inclusion	Articles published in English	This criterion was set due to the language capabilities of the review team and to ensure a comprehensive understanding of texts
	Exclusion	Non-English publications	Language limitations might have impeded accurate interpretation of data and findings

## 3. Results

Table 3 shows the baseline variables assessed across the included papers.^[15–24]^ Zhang et al^[15]^ highlight the crucial role of GAI, such as ChatGPT, in medicine, emphasizing the need for addressing trust, privacy, and safety. Martin-Sanchez et al^[16]^ contribute by developing a competency framework for personalized precision medicine, which outlines necessary skills and training across various healthcare profiles. Lopes et al^[17]^ document the rise of AI in HPE through a bibliometric analysis, noting its significant impact on enhancing clinical competencies and patient education. Lazarus et al^[18]^ provide a critical perspective on AI in anatomy education, identifying challenges and recommending improvements in diversity and transparency in AI applications. Talan et al^[19]^ explore AI educational research trends, emphasizing the dominance of intelligent tutoring systems and machine learning. Masters et al^[20]^ address the ethical considerations in AI usage in HPE, focusing on data privacy, bias, and transparency. Clausmann et al^[21]^ discuss the dual potential of large language models in medical education, advocating for a balanced approach to maximize benefits and minimize risks. Gilson et al^[22]^ evaluate ChatGPT’s performance in medical licensing exams, supporting its utility as an educational tool for exam preparation. Preiksaitis et al^[23]^ explore the opportunities and challenges of GAI in medical education, stressing the need for integrity and accuracy in academic resources. Lastly, Das et al^[24]^ validate the effectiveness of ChatGPT in answering microbiology questions, confirming its role as a valuable learning assistant.

**Table 3:** Demographic variables assessed across the included papers.

Author(s)	Year	Study design	Key findings
Zhang et al^[15]^	2023	Narrative Review	Generative AI, such as OpenAI’s ChatGPT, plays a crucial role in medicine and healthcare by generating various content types. Key issues discussed include trust, veracity, clinical safety, privacy, and opportunities for AI-driven conversational interfaces. The review concludes that generative AI’s role will grow as it becomes better adapted to the medical domain and regulatory frameworks evolve
Martin-Sanchez et al^[16]^	2023	Proposal and Framework Development	A new competency framework for personalized precision medicine is proposed, defining 58 competencies across 5 essential domains and a cross-cutting domain. This framework addresses the training needs of 6 professional profiles in healthcare, suggesting progressive levels of training for optimal performance in personalized medicine
Lopes et al^[17]^	2023	Bibliometric, Descriptive, and Retrospective Analysis	This study analyzed publications related to AI in Health Professions Education from 1990 to 2023. It found that AI’s adoption in healthcare education is increasing, particularly in enhancing clinical competencies and attitudes. AI also plays a significant role in patient education, contributing to health literacy and healthcare management. The study underscores the growing impact of AI in healthcare education
Lazarus et al^[18]^	2022	Critical Analysis	The study explores the integration of AI into anatomy education, highlighting 5 main tensions: human variations, healthcare practice, diversity and social justice, student support, and student learning. It suggests that while AI offers opportunities, it also comes with significant challenges that need careful management. Recommendations include enhancing transparency, embracing diversity among AI developers, and incorporating anatomical variations and uncertainties, all aimed at improving the use of AI in anatomy education
Talan et al^[19]^	2021	Bibliometric Analysis	This bibliometric study investigates the use of AI in education through the Web of Science database, identifying 2686 publications primarily from the USA. Key findings include the prominence of institutions like Carnegie Mellon University and researchers such as Vanlehn, K. and Chen, C.-M. in AI educational research. The analysis highlights the frequent use of terms like “intelligent tutoring systems” and “machine learning,” indicating significant trends in AI application in higher education contexts
Masters et al^[20]^	2023	Ethical Guide	The guide addresses the ethical implications of AI in Health Professions Education (HPE), focusing on issues such as data gathering, privacy, consent, bias, and transparency among others. It aims to prepare HPE teachers and administrators to understand and manage these ethical challenges effectively, ensuring that AI applications in HPE adhere to crucial ethical principles. The guide also suggests proactive measures to deal with potential ethical dilemmas in the use of AI in HPE
Clausmann et al^[21]^	2023	Systematic Overview	Clausmann et al explore the potentials and limitations of large language models (LLMs) like ChatGPT in clinical practice, medical research, and medical education. They discuss how LLMs can democratize medical knowledge, improve accessibility, and actively engage in medical education, yet caution about risks of misinformation and scientific misconduct due to issues like lack of accountability and transparency. This dual potential underscores the need for careful integration of LLMs in health professional education to harness their benefits while mitigating risks
Gilson et al^[22]^	2023	Comparative Performance Evaluation	This study evaluated ChatGPT’s ability to respond to medical licensing examination questions, comparing its performance with other models like GPT-3 and InstructGPT. ChatGPT showed varying accuracies across different datasets but generally outperformed InstructGPT. The findings highlight ChatGPT’s capability to provide logical justifications and incorporate relevant information, suggesting its potential use as an educational tool in preparing students for exams like the USMLE. This aligns with the review’s interest in using generative AI to personalize and enhance learning experiences in health professional education, particularly in high-stakes testing scenarios
Preiksaitis et al^[23]^	2023	Scoping Review	Preiksaitis et al conducted a scoping review to identify the opportunities and challenges of generative AI in medical education. They found themes indicating generative AI’s potential for self-directed learning, simulation scenarios, and academic support. However, concerns about academic integrity and data accuracy were also prominent. The study proposes critical areas for future research, including developing critical evaluation skills, rethinking assessment methodologies, and exploring human-AI interactions. These findings are essential for understanding how generative AI can be tailored to enhance personalized learning and address challenges in health professional education
Das et al^[24]^	2023	Empirical Analysis	Das et al assessed ChatGPT’s ability to answer microbiology questions based on a competency-based curriculum. ChatGPT demonstrated a high level of accuracy in answering both first- and second-order questions, although there was variability across different topics. The study suggests that while ChatGPT can be an effective tool for automated question-answering in microbiology, continuous improvements are necessary. This supports the potential of generative AI as a personalized learning assistant in health professional education, capable of providing accurate responses and adapting to various educational needs

Zhang et al^[15]^ provided insights into the potential uses of generative AI in education, asserting its adaptability to not only general higher education but also medical education across undergraduate, postgraduate, and continuing medical education levels. The implications extended to patient and public health education as well, although medical education was not the exclusive focus of their discussion. A theme issue for JMIR Medical Education highlighted in this investigation garnered considerable attention with its focus on “ChatGPT and Generative Language Models in Medical Education.” This publication attracted numerous submissions, a testament to the growing interest in generative AI’s applications in the educational sphere, particularly in medical and professional education.

In a separate study, Martin et al^[16]^ delved into the competencies required for personalized precision medicine (Table 4). They advocated for a comprehensive framework encompassing a variety of skills, from grasping health determinants to mastering biomedical informatics and practical applications such as pharmacogenetics and AI. The aspect of participatory health was underscored, with patient communication and involvement identified as key components. Bioethics also received attention, with an aim to navigate the ethical landscape particular to precision medicine. The researchers pointed to the importance of transversal competencies, including management and cross-disciplinary collaboration, as indispensable in this field.

**Table 4:** Themes emerged from the review.

Theme	Pointer	Details	Source
Generative AI in Education	Adaptability to Medical Ed.	Generative AI, like ChatGPT, is adaptable to various levels of medical education and can be extended to patient and public health education.	Zhang et al^[15]^
Medical Education Publications	Growing Interest	The JMIR theme issue on generative language models in medical education attracted a range of submissions, indicating a significant interest in the topic.	
Competency Framework for Personalized Precision Medicine	Determinants of Health	– Molecular to psychosocial disease mechanisms	Martin et al^[16]^
	Biomedical Informatics	– Data distinction, management, privacy, and analysis	
	Practical Applications	– Omics technologies, biomarkers, pharmacogenetics, AI, and genetic counseling	
	Participatory Health	– Patient involvement and communication skills	
	Bioethics	– Ethical principles and data handling in precision medicine	
	Transversal Competencies	– Management, leadership, and cross-disciplinary collaboration	

On the front of AI in health professional education, Alves et al^[17]^ provided a thorough examination (Table 5). The results of the bibliometric analysis reveal a significant and progressive impact of AI on research, particularly noted by an upsurge in its application to surgical education post-2018. AI’s role, as defined by Alves et al,^[17]^ encompasses the creation of machines or software capable of tasks that typically require human intelligence, including learning and problem-solving. The suite of AI technologies encompasses machine learning, deep learning, natural language processing (NLP), and computer vision, which contribute to potential benefits such as personalized learning, intelligent tutoring, virtual simulation, and automated grading. Importantly, AI’s contribution to research is not just functional but also analytical, with visualization techniques providing insights into the scientific structure and evolution of AI within healthcare education.

**Table 5:** Benefits and challenges pertaining to the Integration of GAI in PHE.

Theme	Pointer	Details	Source
AI in Health Professional Education	Definition of AI	AI involves creating machines or software that can perform tasks requiring human intelligence, such as learning and problem-solving	Alves et al^[17]^
	AI Technologies	Includes machine learning, deep learning, NLP, and computer vision	
	Potential Benefits	Personalized learning, intelligent tutoring, virtual simulation, automated grading	
	Research Impact	Visualization techniques help comprehend the scientific structure and development of AI in healthcare education	
Bibliometric Analysis	Study Characteristics	Bibliometric, descriptive, and retrospective study using PubMed data from 1990 to 2023	
	Search Strategy	Used VOS viewer for term extraction and trends visualization	
	Literature Volume	576 relevant references found, including clinical trials and meta-analyses	
	Trends in Usage	Common in medical fields and educational levels, significant uptake in surgery after 2018	
Ethical and Responsible AI Use	Ethical Considerations	Need for ethical, responsible AI design considering bias, privacy, and transparency	
	Potential Challenges	Disadvantages may arise for some learners and within certain contexts	Lazarus et al^[18]^
Tensions in AI Integration	Anatomical Education Challenges	Human variations, healthcare practice, diversity, and social justice issues	
	Student Support & Learning	Uncertainties in student support and learning when integrating AI	
Recommendations for AI Use	Transparency and Diversity	Enhanced transparency, AI developer diversity, and inclusion of anatomical variations	
	Educator and Curriculum Development	Educator awareness of AI benefits/limitations and “AI-free” curricular time	
	Extending Human Capacities	Using AI to augment rather than replace human capacities in education	

The bibliometric study itself was descriptive and retrospective, using PubMed data spanning from 1990 to 2023. The search strategy involved the use of VOS viewer for term extraction and the visualization of trends. From this analysis, 576 relevant references were identified, encompassing clinical trials and meta-analyses. These references indicate a broad application of AI across various medical fields and educational levels, with a marked increase in its application within the domain of surgery since 2018. The findings underscore the growing importance of AI in enhancing research and education within the healthcare sector.

However, the integration of AI was not without its ethical dilemmas. Challenges such as potential biases, privacy concerns, and the need for transparency were highlighted, with a call for an ethical and responsible approach to AI design and implementation (Table 5). Lazarus et al^[18]^ further discussed these challenges, particularly in the realm of anatomical education, where the diversity of human variation and social justice issues were at play. They stressed the importance of diversity among AI developers and a mindful approach to student support when incorporating AI into learning environments, advocating for strategies that would enhance transparency and accommodate a wide range of learning needs.

Talan et al^[19]^ conducted a study that delved into the central concepts of AI research, unearthing core keywords such as “AI,” “intelligent tutoring systems,” “machine learning,” “deep learning,” and “higher education” (Table 6). The researchers found these terms to hold high centrality within the literature – an indicator of their pivotal role and frequent appearance in this field. Moreover, alongside these popular keywords emerged additional hot topics including: data science; learning analytics; computer-based learning – evidence of how expansive and dynamic research areas within AI truly are.

**Table 6:** Central themes in GAI research and ethical considerations in PHE.

Theme	Pointer	Details	Source
Central Concepts in AI Research	Keyword Centrality	AI, intelligent tutoring systems, machine learning, deep learning, higher education are central keywords	Talan et al^[19]^
	Popular Topics	Data science, learning analytics, computer-based learning, educational data mining	
AI in Health Professions Education	Current Benefits	PHE currently benefits from AI advancements	Ken et al^[20]^
	Future Potential	AI set to offer more benefits to PHE in the future	
Ethical Considerations in AI	Ethical Focus	Focus on ethics in the use of AI systems within PHE	
	Ethical Issues	Data gathering, anonymity, privacy, consent, data ownership, security, bias, transparency, responsibility, autonomy, beneficence	
	Ethical Guidance	Guide provides concepts, their importance, coping strategies, and suggests further reading	
	Proactive Measures	Encouragement for PHE teachers and administrators to be proactive in ethical AI usage	
Educational strategies	LLMs as teaching assistants	LLMs can provide summaries, presentations, translations, explanations, and guides in customizable formats, enhancing personalized education. They are suitable for creating interactive simulations, like patient history taking practices for medical students	Clusmann et al^[21]^
Critical thinking concerns	Impact on student skills	LLMs might negatively affect students’ critical thinking and creativity by providing easy answers, risking the externalization of factual knowledge and medical reasoning. Transparent regulation and differentiation between LLM-generated content and student work are necessary	
Responsible use of LLMs	Education & interaction guidelines	Essential to establish guidelines for LLM use to prevent misuse. Students in medical education should receive an introduction to LLMs, learning about biases, limitations, and proper prompt engineering to avoid misinformation	
AI performance on exams	AMBOSS users comparison	ChatGPT scored in the 30th percentile on Step 1 without Attending Tip and 66th with it; on Step 2, 20th and 48th percentiles, respectively. Accuracy decreased with question difficulty	Gilson et al^[22]^
	NBME vs AMBOSS performance	ChatGPT performed better on Step 1 than Step 2 and better on NBME questions than AMBOSS for both levels. Outperformed GPT-3 and InstructGPT on all data sets	
	Logical use of information	ChatGPT provided logical explanations, using internal information for most responses and external information significantly more often in correct responses	

Ken et al^[20]^ concentrated on applying AI in PHE. Their report acknowledged PHE had already started reaping rewards from AI progress, enhancing diverse facets of the educational process through current applications (Table 6). They found significant potential for AI to extend these benefits further in future; consequently, they set expectations for a more comprehensive integration impending ahead within PHE. Ken et al^[20]^ also made ethical considerations in the use of AI systems within PHE a focal point of their research. They underscored an array of pertinent ethical issues to deploying AI in educational settings: data gathering concerns, anonymity and privacy matters, consent-related challenges; ownership disputes over collected information; security threats – both internal and external – bias instances that could emerge from algorithmic processes or human interactions alike, transparency questions about decision-making procedures involved with these technologies – all underpinned by responsibility aspects for both developers as well as users.

**Figure 3.**
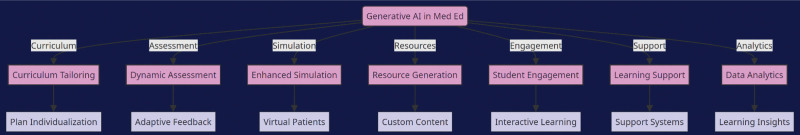
Different facets of GAI that influence medical education. GAI = generative artificial intelligence.

Clusmann et al^[21]^ contributed to this discourse by specifically addressing the impact of LLMs in medical education. Their insights into the potential erosion of critical thinking skills due to the facilitation offered by LLMs resonate with the prevalent ethical apprehensions expressed by other studies. The concurrence on the necessity of transparent regulations and informed usage of AI tools indicates a shared understanding of the delicate balance between benefit and risk in AI adoption (Fig. 3). The prospect of AI as a supplementary educational resource has been contemplated, with the potential to individualize learning trajectories and complement the role of human educators. This approach to AI-assisted education suggests a paradigm where AI acts as a dynamic facilitator of the educational process, adaptable to the unique educational needs of each learner.^[22]^

Preiksaitis et al^[23]^ noted both the potential and limitations of GAI in standardized exams, reflecting the dualistic nature of GAI as an educational aid and obstacle. The research indicated GAI’s effectiveness in certain areas like cardiology and neurology, while also pointing out inconsistencies, such as those observed in Taiwan Family Medicine Board Exam (Table 7). These findings underscored GAI’s variable performance across different domains. The collective research underscored GAI’s role in personalizing education and offering innovative learning strategies, yet empirical evidence for its effectiveness in self-study and exam preparation was scarce, suggesting a need for more applied research. GAI’s support in writing, language assistance, and literature reviews was especially beneficial for non-native English speakers, a consistent finding across the other studies that we assessed. However, concerns such as potential academic dishonesty, misinformation, and the undermining of critical thinking skills were universally recognized, emphasizing the necessity for clear academic policies and updated assessment strategies. The research stressed the importance of responsible GAI use within educational frameworks.

**Table 7:** Multifaceted impacts of GAI on medical education.

Theme	Pointer	Details	Source
Enhancing test performance	Standardized exam success	Generative AI has shown promise in standardized exams like cardiology and neurology but struggled with some, like Taiwan Family Medicine Board Exam	Preiksaitis et al^[23]^
	Self-directed learning	AI could aid in self-study and exam preparation, but research on practical applications is limited	
Innovating learning strategies	Personalized education	AI’s adaptability may allow for personalized learning experiences with tailored plans and feedback	
	Communication skills	AI could be used to simulate difficult conversations, improving communication in emergency medicine	
	AI-generated images	Enhances case-based learning and medical humanities without privacy or copyright issues	
Writing and research assistance	Language support	AI can assist non-native English speakers in writing and translating materials	
	Literature Reviews	AI may aid in summarizing literature but with caution due to the potential creation of false references	
Potential limitations and risks	Academic integrity	AI misuse could lead to cheating and authorship misrepresentation, requiring clear academic policies	
	Accuracy and dependability	AI’s knowledge is limited by training data, and it may produce outdated or incorrect information	
	Overdependence and assessment	Heavy reliance on AI could weaken critical thinking; assessments might need updating to remain valid	
Expert validation & scoring	Expert-validated content with consistent scoring indicates AI’s reliability in educational settings	Reinforces the potential of AI as a dependable tool for learning and assessment	Das et al^[24]^
Interaction & statistical analysis	Effective engagement and robust statistical analysis demonstrate AI’s potential in interactive and analytical educational roles	Supports the integration of AI into educational methodologies for enhanced learning experiences	
Overall & topic-wise performance	High mean scores overall with some variability across topics suggest AI’s proficiency with room for improvement in certain areas	Indicates the effectiveness of AI in education while highlighting areas for targeted improvement	
Score distribution & performance variation	Consistent performance across different question types but significant variation across topics points to AI’s strengths and limitations	Suggests a need for a nuanced approach to implementing AI in diverse educational content areas	

Das et al^[24]^ added the perspective of expert validation and consistent scoring, endorsing GAI’s reliability in educational settings and echoing the need for a balanced approach in its application, calling for robust validation and risk mitigation measures.

## 4. Discussion

Collectively speaking, the studies infer a readiness among healthcare professionals for AI integration, underscored by positive attitudes toward ongoing education and technological engagement. They are similar in recognizing the potential of AI to enhance HPE, the need for ethical vigilance, and the importance of maintaining human elements in education. They diverge in focus, with Martin et al^[16]^ detailing a competency framework; Alves et al^[17]^ defining AI and its educational potential and analyzing literature trends; and Lazarus et al^[18]^ underscoring ethical and responsible AI use. The degree of similarity and dissimilarity among the studies is thus context-dependent, predominantly unified around the central theme of AI as a transformative element in HPE, yet distinguished by each study’s specific emphasis on demographics, competencies, technological potential, or ethical considerations.

Talan et al^[19]^ and Ken et al^[20]^ both explore the landscape of AI in education, albeit from different angles. Talan et al^[19]^ identify key themes and terminologies in AI research, indicating a diverse and expanding field with emerging areas such as data science and learning analytics. This conceptual mapping^[19]^ suggests a field in flux, with AI research touching numerous facets of higher education. Ken et al^[20]^ also acknowledge the changing terrain of AI in education, specifically within PHE, by anticipating a deeper future integration. Their work^[20]^ focuses on the ethical ramifications of this integration. While both studies^[19,20]^ share a common understanding of the evolving role of AI, Talan et al^[19]^ concentrate on AI’s academic discourse and Ken et al^[20]^ are more concerned with practical implications and ethical stewardship in educational practice.

**Figure 4.**
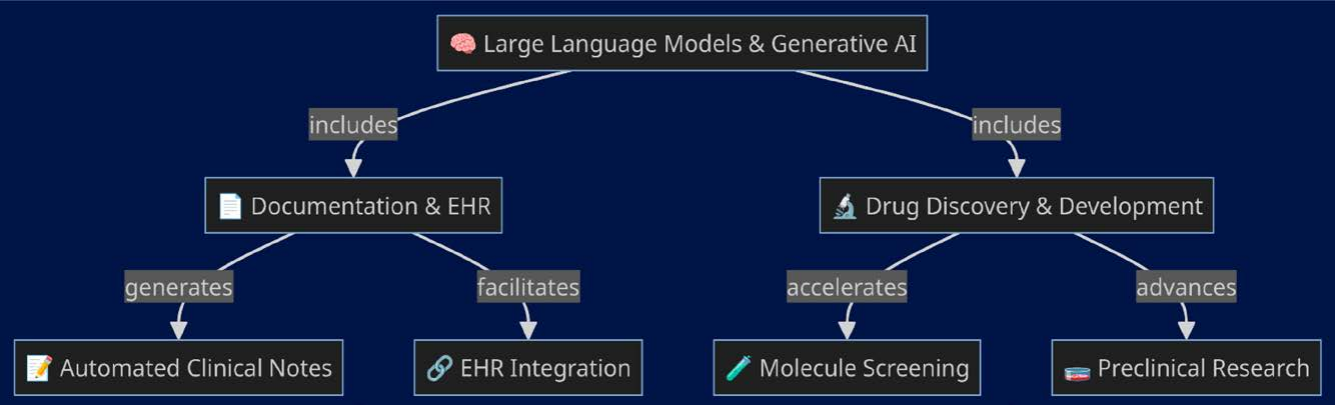
LLM’s and GAI’s accelerative effect on documentation and drug discovery. GAI = generative artificial intelligence, LLMs = large language models.

In the realm of higher education and standardized testing performance, the capabilities of GAI systems have been scrutinized, particularly in the context of medical education (Fig. 4). These GAI entities have been demonstrated to possess the capacity to achieve moderate levels of accuracy in various assessments pertinent to medical training.^[25]^ For instance, in evaluations modeled after resuscitation courses endorsed by the American Heart Association (AHA), AI systems have registered scores within the moderate percentile range, albeit below the established proficiency benchmarks. These scores, while not qualifying for certification, do exhibit the GAI’s ability to generate pertinent and comprehensive explanations, occasionally surpassing the detail found in standardized answer keys.

Further examination into AI performance on medical licensure preparatory materials, specifically those aligned with the US Medical Licensing Examination (USMLE), has revealed that AI can achieve a baseline comprehension sufficient to match the passable standards expected of a medical student in their third year of study. GAI’s narrative coherence across its responses was particularly noted, suggesting an adeptness at contextual understanding.^[26]^

Adapting learning experiences to individual requirements and different stages of professional development is a skill that this technology excels at, and it is becoming more and more crucial in diverse educational contexts.

Our review demonstrates the potential of generative AI to be used in patient and public health education by highlighting its useful application at various medical education levels. This wide range of applications highlights a move toward more adaptable educational frameworks that can take into account a variety of students and learning settings.

The results of our assessment point to the need for additional research in order to improve AI applications in educational settings and make sure they are flexible and efficient. In order to assess the effects of AI-enhanced learning methodologies on educational outcomes and provide students with the skills necessary to navigate the complexity of contemporary healthcare contexts, empirical research is especially crucial.

Recent investigations on the possibilities and constraints of sophisticated GAI as tools for assisting in clinical decision-making have shed light on these tools’ capabilities. Notably, when evaluated against a variety of clinical settings, these models have proven adept at reaching final diagnoses.^[27]^ Nonetheless, it seems that the initial differential diagnosis generation is a difficult domain, since these algorithms frequently exhibit subpar performance. When these LLMs are applied to clinical management tasks, differences in performance are found. These models can provide evaluations and recommendations that are comparable to the competency level of early medical trainees, even when addressing simple cases.^[28]^ However, as cases become more complex, they become less successful, particularly when dealing with illnesses that are diverse and involve multiple systems. In these circumstances, important details can be missed, which would lead to an insufficient case handling.^[27]^

LLM’s ability to condense clinical interactions into coherent narrative summaries is a noteworthy strength. Nevertheless, there has not been enough consideration of important demographic elements like patient age in the clinical reasoning process.^[29]^ Furthermore, these models show notable shortcomings when addressing uncommon or complex clinical situations that frequently call for expert judgement and are the topic of scholarly discussion in the medical community.^[30]^ Furthermore, GAI’s tendency to confabulate – to provide answers that, although comprehensible to laypeople, are inherently flawed – is one of the main causes for concern.^[29–32]^ This propensity may result in unsuitable treatment regimens and inaccurate diagnoses, which has repercussions for the delivery of healthcare. These mistakes show how dangerous it is to use these models without caution and how important human judgement is when making complicated therapeutic decisions.

The findings from the scoping review by Preiksaitis et al^[23]^ resonate with the themes presented in the narrative review and other cited studies, highlighting both the potential applications and challenges of generative AI in medical education. Like Khan et al,^[33]^ they acknowledge the significant impact and rapid integration of AI technologies like ChatGPT in medical education and clinical management, while cautioning against overreliance and the limitations of AI. Both sets of findings underscore the importance of using AI as an assistive tool rather than a replacement for human expertise.

Abd et al^[34]^ expand on the transformative possibilities of LLMs in medical education, similar to the narrative review’s discussion on the potential of generative AI for personalized learning experiences. However, Abd et al^[34]^ also delve deeply into the challenges, including algorithmic bias and misinformation, which mirror the ethical and responsible AI use concerns raised by Lazarus et al^[18]^ in the narrative review. These parallels draw attention to the crucial need for critical evaluation and responsible AI integration in educational settings.

Karabacak et al^[35]^ propose that AI and generative language models (GLMs) offer significant opportunities for enhancing medical education, aligning with the narrative review’s insights into AI’s role in competency development and the provision of immersive learning environments. However, they also highlight the need for content quality assurance, bias management, and ethical considerations, echoing the narrative review’s emphasis on the complexity of effectively integrating AI into medical education. The potential applications of conversational GAI in educational settings, especially within medical training, have been posited to extend beyond mere knowledge assessment. GAI’s ability to furnish on-demand, interactive learning support has been suggested to potentially bolster critical thinking, problem-solving aptitude, and reflective learning among students.^[36]^

### 4.1. Limitations

The review and broader literature reveal a significant gap in empirical studies featuring well-defined methodologies and systematically documented outcomes, highlighting a critical shortcoming in this emerging field. Given that GAI is in its infancy, there is a scarcity of experimental trials or case–control studies, which presents a challenge in assessing GAI’s application across varied medical educational domains. This issue is pressing not only in terms of GAI’s performance but also in regard to ethical considerations. Furthermore, relying on opinion-based studies to formulate policies and recommendations could lead to the premature adoption of a technology that may not deliver expected outcomes. This is particularly relevant as GAI is being heralded as a transformative tool for personalized health education across disciplines, including medicine, pharmacy, dentistry, and physiotherapy.

### 4.2. Recommendations pertaining to the usage of GAI

In light of the synthesis of current research, it is essential to guide future investigations toward the development and evaluation of curricular advancements that incorporate ethical considerations of GAI within PHE.

1. *Embrace generative AI for educational adaptability*: Educational institutions should embrace the adaptability of generative AI to enhance medical education at various levels. By integrating AI, curricula can be tailored to meet the diverse educational needs of students across different stages of learning, including patient and public health education.
2. *Leverage AI to address competency framework for personalized precision medicine*: It is recommended to incorporate AI tools in teaching determinants of health, from molecular to psychosocial factors, and to use AI to facilitate understanding in biomedical informatics, including data management and privacy. Generative AI can support practical applications such as omics technologies and pharmacogenetics, and enhance participatory health by improving patient involvement and communication skills. Ethical considerations, particularly in data handling, should be integrated into health professional education.
3. *Integrate AI into health professional education strategically*: AI’s potential to personalize learning experiences, intelligent tutoring, and virtual simulations can be harnessed to improve health professional education. However, this should be done with a clear understanding of AI’s capabilities, and educators should be aware of both the benefits and limitations of AI applications.
4. *Promote ethical and responsible ai use*: Develop and enforce guidelines for ethical and responsible AI use. Considerations should include addressing bias, ensuring privacy, maintaining transparency, and promoting fairness to ensure that all learners benefit equally from AI technologies.
5. *Develop educational strategies incorporating AI*: Educators should consider using generative AI, such as LLMs, as teaching assistants to create interactive simulations and customizable educational content. However, it is critical to establish guidelines for their use to prevent reliance on AI that may affect critical thinking skills and creativity.
6. *Foster critical thinking despite AI assistance*: While AI can assist in standardized test preparation and performance, educators should ensure that reliance on AI does not replace the development of critical thinking and problem-solving skills. Educational assessments may need to be revised to reflect this balance.
7. *Innovate learning strategies with AI*: AI can be used to personalize education and improve communication skills through simulated interactions. Additionally, AI-generated images can enhance case-based learning, and AI can also provide language support for non-native English speakers.
8. *Address potential limitations and risks*: Institutions must be vigilant about academic integrity, ensuring that AI is not misused for cheating or misrepresentation. Furthermore, the accuracy of AI-generated content should be monitored, and overdependence on AI should be avoided to ensure that critical thinking skills remain sharp.
9. *Involve experts in validation and scoring*: Incorporate expert validation of AI-generated content to ensure its reliability and accuracy. This will support AI as a dependable tool for education and assessment.
10. *Evaluate AI’s role through interaction and statistical analysis*: Engage in robust statistical analysis to gauge the effectiveness of AI in interactive educational roles. This will help in refining AI integration into educational methodologies.
11. *Monitor AI performance for continuous improvement*: Keep track of AI performance across different educational topics and content areas. This will help in identifying areas where AI needs improvement and ensure that its integration into education is effective and enhances learning experiences.

## 5. Conclusions

Our findings illuminate a gender-balanced healthcare workforce, inherently inclined towards continuous professional development and the utilization of digital tools. They also underscore the necessity for an all-encompassing educational framework that incorporates a broad range of skills vital in precision medicine; this underlines not only patient engagement but also bioethics’ significance. The integration of AI in PHE highlights significant ethical challenges. A strong emphasis is placed on the necessity for an ethically designed approach and diverse development teams, recognizing that these issues are paramount. The review also asserts that GAI harbors tremendous potential in customizing PHE; however, it underpins this endorsement with a crucial stipulation – the pressing demand for ethical structures and varied developer teams to tackle bias and equity issues within educational GAI applications.

## Acknowledgments

The authors extends the appreciation to the Deanship of Postgraduate Studies and Scientific Research at Majmaah University for funding this research work through the project number (R-2024-1184).

## Author contributions

**Conceptualization:** Mohammed Almansour, Fahad Mohammad Alfhaid.

**Data curation:** Mohammed Almansour, Fahad Mohammad Alfhaid.

**Methodology:** Mohammed Almansour.

**Project administration:** Mohammed Almansour.

**Resources:** Mohammed Almansour.

**Supervision:** Mohammed Almansour.

**Validation:** Mohammed Almansour, Fahad Mohammad Alfhaid.

**Writing – review & editing:** Mohammed Almansour.

**Formal analysis:** Fahad Mohammad Alfhaid.

**Visualization:** Fahad Mohammad Alfhaid.

**Writing – original draft:** Fahad Mohammad Alfhaid.

## Appendix A

Figures, Tables.
